# University Developments

**Published:** 1918-01-19

**Authors:** 


### The Hospital

The Workers' Newspaper of Administrative Medicine and Institutional Life, Administration, National Insurance and Health.

No. 1650, Vol. LXIII. SATURDAY, JANUARY 19, 1918. PRICE ONE PENNY.

### UNIVERSITY DEVELOPMENTS.

In spite of certain recent official activities it would hardly be accurate to say that educational enterprises have come into the front rank of public favour and recognition. But if they have not actually reached this position, they threaten to assert their claims to it. In the meantime, the competition of the overmastering issue of the war has pushed them, comparatively speaking, into the background, where, at least so their friends believe, they may be content to wait with the conviction that the hour of their promotion is at hand. For our own part we share this conviction, or, if this is a somewhat excessive statement, we may express the hope that time will justify it. So far as it can be turned in the educational direction the general attention is chiefly concerned with what may be called the lower levels, or, with equal accuracy, the foundation scheme. It is here, principally, that Parliamentary ambitions express themselves, and the arrangement is a most seemly one, for such a scheme is the first essential and concerns the country and the nation as a whole. It is the first rung of the ladder, and obviously it must be made secure prior to any attempt to construct those other steps which may in due course lead capable and ambitious youth from the school-house to the University. We may agree that the enterprise is a laudable one, though not necessarily for the reasons which find special favour in the eyes of many of those who direct the Universities. These, quite naturally, magnify the end, and probably they are not altogether wrong, even though they forget the discipline of the ascent and fail to recognise academic values other than those which flow from professorial prelections. In the meantime it is of interest to note that with the proposed improvements in elementary, and to some extent at least in secondary, education, it is found possible to associate improvements or developments in the University scheme of things. Like other organisations in these restricted days, the Universities have fallen upon evil financial times, and in certain of them not a few of the teachers are suffering severe financial hardship. If you scatter the pupils you take away the fees, and even literature may not be cultivated without the aid of "a little oatmeal." Yet, in spite of the scarcity of the times, two of the Northern Universities have managed during the last few months to establish each a new professorship, and we know that these institutions, acting on a sound principle, do not engage on such enterprises unless there is a certain and sufficient endowment for each academic chair. Probably in each of these instances the necessary funds have arrived from some source which took origin prior to the war. But, in any event, here is the impressive fact that, while in certain other quarters teachers are driven to seek some less spiritual occupation, two of the Scottish Universities are able to enlarge their academic equipment. In Glasgow it is Ophthalmology; in Edinburgh, Tuberculosis.

With many others interested in medical education we heartily join in congratulating the two Northern Universities, and we wish a large success to the new developments. With all the influences which have led to these we cannot profess to be acquainted, but in a broad and general sense such developments must be regarded as the outcome of the ever-extending borders of knowledge, and of the necessity which is placed upon each department to assert itself, lest haply it be obscured by its more vocal competitors. This is a somewhat mundane motive to propose, but none the less it is a very real one. Not only the student world, but also the larger world which is outside the cloisters, has to listen to many appeals to give heed and attention, and the supply of these qualities is not an unlimited one. In the very nature of things a voice which is subdued, and still more one which is silent, will suffer a heavy handicap and may largely escape even recognition. Hence in the academic scheme a subject which has no official sponsor must appear of relatively insignificant importance, and its virtues and attractions and merits will escape the attention they deserve. It is here that the special professor comes in. His duty is to justify his existence by keeping the subject of his chair prominent in season and even out of season. Unless he is capable of this assertion he will fail in his main purpose. Human nature being what it is, and academic competition being what it is, there is a necessity to be up and doing, and the condition is an excellent one, for it has stimulating values. It may be that in satisfying it the individual professor is somewhat inclined to over-emphasis, for not everyone is equipped with so detached and impartial a view of the whole field of knowledge that he can place the subject which he cultivates in its exact and just perspective. Besides, there is the competition and pressure of neighbours and rivals. Each one of these is vocal, and the example invites to imitation or even compels it. After all, there is nothing like leather, and University professors, even as less distinguished mortals, must recognise the maxim. Far be from us the suggestion that this is their sole duty. Nevertheless it is one of their duties, and they have to recognise it among the purposes of their existence. The most solid justification for this enterprise is fruitful work or effective teaching, or, still better, both of these achievements. But some measure of worldly wisdom is also necessary. The professors have wares to sell, and they must make them known to the constituency. There is competition in the market, and the silent cannot hope for buyers. Even the academic world is a busy one, and gives attention only to the emphatic. The test is not proclaimed loudly, for under the play of a large charity every professor is deemed to be learned just as every magistrate is affirmed to be worshipful. None the less the verdict is framed even though the court delivers it *in camera*, and there is a shaking of heads which is never publicly advertised. It is in such considerations as these that is to be found the justification for the creation of new professorships as special subjects take on an enlarged scope and the older chairs meet them with imperfect recognition. Being late to arrive such subjects need particular emphasis to bring them into their due order, and if the professors concerned with them magnify their office the error will be in the right direction. Nor will they lack examples among their colleagues. The performance means some sacrifice of individual completeness and a degree of one-sided development. But this is true of the division of labour generally and matters little so long as through it " the world is more and more."

